# Contact Lenses Incorporating Methotrexate-Loaded Chitosan/Hyaluronic Acid Nanoparticles

**DOI:** 10.3390/biomimetics11080532

**Published:** 2026-08-01

**Authors:** Sofia Vale, Sara F. M. Senra, Sérgio R. S. Veloso, Elisabete M. S. Castanheira, Madalena Lira

**Affiliations:** 1 Physics Centre of Minho and Porto Universities (CF-UM-UP), University of Minho, Campus de Gualtar, 4710-057 Braga, Portugal; pg50962@alunos.uminho.pt (S.V.);; 2 LaPMET Associate Laboratory, University of Minho, Campus de Gualtar, 4710-057 Braga, Portugal; 3 CINBIO, University of Vigo, 36310 Vigo, Spain; sergiorafael.dasilva@uvigo.gal

**Keywords:** therapeutic contact lenses, drug delivery, methotrexate, nanoparticles, chitosan, hyaluronic acid, biomimetic materials

## Abstract

Nanoparticle-laden contact lenses (CLs) represent a promising strategy for ocular drug delivery. Nanocarriers built from hyaluronic acid and chitosan offer a biomimetic alternative to synthetic polymers, by combining the lubricating property of hyaluronic acid with the mucoadhesive property of chitosan. However, achieving sustained drug release without compromising lens properties remains challenging, and the influence of lens material and replacement modality is unclear. This study evaluated methotrexate (MTX)-loaded chitosan/hyaluronic acid (CS/HA) nanoparticles incorporated into silicone hydrogel CLs with different replacement modalities, assessing their effect on drug release kinetics. Daily replacement lenses (Senofilcon A and Delefilcon A) released 26 ± 4% and 33 ± 5% of MTX after 24 h, respectively. The monthly lens Lehfilcon A showed slower diffusion-controlled release, with only 15 ± 2% released at 24 h and a 64 ± 3% cumulative release after 31 days (*p* < 0.01). Nanoparticle incorporation improved drug retention and reduced initial drug loss compared with lenses loaded with only MTX. Monthly lenses demonstrated sustained delivery potential, supporting prolonged ocular therapy, while daily lenses may be better suited for short-term treatment. These findings reinforce the value of bioinspired nanocarriers, mimicking natural retention mechanisms of the ocular surface, for next-generation therapeutic CLs.

## 1. Introduction

Topical eye drops remain the most commonly prescribed route for ocular drug administration; however, their therapeutic efficiency is markedly limited by rapid tear turnover, nasolacrimal drainage, and low corneal permeability. It is estimated that less than 5% of an instilled drug dose effectively penetrates intraocular tissues, resulting in poor bioavailability and the need for frequent dosing regimens, which may compromise patient adherence and therapeutic outcomes [1,2].

In recent years, contact lenses (CLs) have emerged as promising platforms for ocular drug delivery due to their prolonged residence time on the ocular surface and their ability to provide sustained drug release compared with conventional eye drops. Drug-eluting CLs may significantly increase drug bioavailability while reducing dosing frequency, offering a potential alternative for the management of chronic ocular diseases [3,4].

Beyond drug delivery alone, the broader landscape of contact lens technology is rapidly evolving toward intelligent, multifunctional wearable platforms capable of continuous biosensing, diagnostics, and integrated therapeutic feedback [5,6]. While these theranostic and sensing-enabled lenses represent an important complementary direction for ocular technology, passive drug-eluting lenses, such as the ones developed in this work, remain a comparatively simpler and more readily translatable strategy for sustained ocular drug delivery, without the added complexity, cost, and biocompatibility challenges associated with embedded electronics. Despite these advantages, achieving controlled and sustained drug release from CLs remains challenging. Conventional drug loading approaches frequently result in burst release profiles and limited drug retention within the hydrogel matrix. To overcome these limitations, nanoparticle-based strategies have been proposed to enhance encapsulation efficiency and modulate release kinetics through diffusion-controlled mechanisms [7].

Chitosan-based nanoparticles have attracted considerable attention in ocular drug delivery owing to their biocompatibility, biodegradability, and mucoadhesive properties, which improve drug retention at the ocular surface. The incorporation of hyaluronic acid further enhances stability, hydration, and ocular compatibility, while promoting favourable interactions with hydrogel contact lens materials [8].

Notably, the choice of these two biopolymers follows a biomimetic rationale: hyaluronic acid is the same glycosaminoglycan naturally present in the tear film and in the ocular mucin layer, where it is responsible for retaining water and lubricating the corneal surface, while chitosan shares some structural similarities with the glycosaminoglycans found in the ocular glycocalyx; however, unlike the typically anionic glycosaminoglycans, chitosan is cationic, which allows it to engage in electrostatic interactions with the negatively charged mucosal surface. By combining these two materials, the resulting nanoparticles essentially aim to mimic, at a smaller scale, the lubrication and retention mechanism that the eye already uses to keep the tear film adhered to its surface, rather than relying on a synthetic carrier with no biological counterpart.

Recent studies exploring therapeutic CLs developed in academic and clinical research settings have demonstrated the feasibility of drug-eluting hydrogel lenses capable of sustained ocular drug delivery, reinforcing their potential as non-invasive therapeutic platforms for chronic ocular diseases [9]. Senra et al. (2025) developed CLs incorporating albumin/hyaluronic acid nanoparticles as a delivery system for 5-Fluorouracil [10]. Their study demonstrated that the nanoparticle-laden lenses could effectively load and release the drug in a controlled manner, highlighting the potential of functionalized CLs as platforms for ocular drug delivery. This approach provides a foundation for evaluating the release kinetics of therapeutics from lenses after immersion in drug solutions.

Methotrexate (MTX), an anti-inflammatory and immunomodulatory agent widely used in autoimmune and inflammatory diseases [11], has also been evaluated for various ocular surface conditions due to its ability to suppress immune responses [12]. However, conventional topical administration suffers from poor ocular residence time and low bioavailability because of tear drainage, lacrimation, and rapid precorneal elimination, which limit therapeutic levels at the ocular surface and require frequent dosing [13,14].

In a recent clinical study, topical MTX was well tolerated when used off-label to treat recalcitrant ocular surface disease, showing minimal cytotoxicity in vitro and improvements in clinical signs of inflammation in patients [12]. Despite this, MTX has been shown to face challenges in ocular delivery such as limited penetration across ocular barriers and potential local irritation, highlighting the need for alternative delivery strategies capable of maintaining sustained therapeutic levels at the ocular surface [12].

Therefore, the present study aimed to evaluate methotrexate-loaded chitosan/hyaluronic acid nanoparticles incorporated into different silicone hydrogel CLs and to investigate how lens material and replacement modality influence drug adsorption and release kinetics under simulated ocular conditions.

## 2. Experimental Section

### 2.1. Materials and Reagents

#### 2.1.1. Contact Lenses

Daily disposable silicone hydrogel CLs (Delefilcon A, Alcon Inc., Fort Worth, TX, USA, and Senofilcon A, Johnson & Johnson Vision Care, Jacksonville, FL, USA) and monthly silicone hydrogel CLs (Lehfilcon A, Alcon Inc., Fort Worth, TX, USA) were used. It should be noted that these are commercial, terminally sterilized products; nanoparticle and drug loading was performed afterwards by soaking, so the drug and nanoparticles were not exposed to the autoclave temperatures used to sterilize the base lens. The CL properties are detailed in Table 1. All the CLs used had a power of −2.50 D to −3.50 D for a slight or negligible effect of the lens thickness [15].

#### 2.1.2. Reagents

Chitosan (448869, low molecular weight, 75−85% degree of deacetylation), tripolyphosphate (TPP), methotrexate (MTX), and hyaluronic acid (HA, 924474, low viscosity, low endotoxin) were obtained from Sigma-Aldrich/Merck (Darmstadt, Germany). Ultrapure water Milli-Q^®^ grade (MilliporeSigma, Burlington, MA, USA) was used for the preparation of all solutions.

### 2.2. Characterization Techniques

The optical properties of the samples were evaluated by ultraviolet–visible (UV-Vis) absorption/transmission spectroscopy, using a Shimadzu UV-3600 Plus UV-Vis-NIR spectrophotometer equipped with an integrating sphere (Shimadzu Corporation, Kyoto, Japan). Fluorescence emission spectra were obtained with a Horiba-Jobin Yvon Fluorolog 3 spectrofluorimeter (Horiba-Jobin Yvon, IBH Ltd., Glasgow, UK).

The average hydrodynamic diameter, polydispersity index, and zeta potential of the nanoparticles were determined by dynamic light scattering (DLS) using a Litesizer™ 500 instrument (Anton Paar GmbH, Graz, Austria). Measurements were performed in triplicate (three independent runs) at 25 °C using a 40 mW semiconductor laser operating at 658 nm and a backscattering detection angle of 175°.

Fourier transform infrared (FTIR) spectra were collected on a PerkinElmer Spectrum Two™ IR spectrometer (PerkinElmer Inc., Waltham, MA, USA) fitted with an attenuated total reflectance (ATR) accessory. Spectra were recorded over the 4000–450 cm^−1^ range with a spectral resolution of 8 cm^−1^ and 32 accumulated scans. Contact lenses were air-dried for 24 h prior to FTIR-ATR measurements.

The morphology of the nanoparticles was examined by scanning transmission electron microscopy (STEM) using a FEI Nova NanoSEM 200 (FEI, Hillsboro, OR, USA) operated at an accelerating voltage of 15 kV. The microscope was equipped with an energy-dispersive X-ray spectroscopy (EDS) detector and an EDAX Pegasus X4M electron backscatter diffraction (EBSD) system and is part of the SEMAT/UM (*Serviços de Caracterização de Materiais, Guimarães, Portugal*) facilities. STEM images were analyzed with ImageJ software (National Institutes of Health, Bethesda, MD, USA, version 1.54r) following image enhancement by local contrast adjustment and brightness optimization prior to nanoparticle selection.

The refractive index and water content of the CLs were measured using a digital refractometer (CLR 12-70, Index Instruments, Huntingdon, UK). Before analysis, excess surface water was carefully removed with absorbent paper while the lenses were held with tweezers, following the procedure previously reported by Lira et al. [18] Transmittance spectra of the CLs were acquired in the 200 to 700 nm wavelength range. Excess surface water was removed from the CLs as previously described, and the lenses were positioned on an appropriate holder, with the convex surface facing the incident light beam.

### 2.3. Preparation of Nanoparticles

For the preparation of the nanoparticles, three stock solutions were prepared: chitosan (CS), tripolyphosphate (TPP), and hyaluronic acid (HA), each at a concentration of 0.1 wt% and with ultrapure Milli-Q water as the solvent. Hydrochloric acid 1 M was added to the chitosan solution to facilitate its dissolution, as chitosan does not dissolve at neutral pH. The concentrations of CS and TPP were varied between the solutions in order to optimize nanoparticle formation (Table S1 in Supplementary Materials). The best formulation was chosen for the subsequent assays.

### 2.4. MTX Encapsulation in CS/HA Nanoparticles

Loading of nanoparticles with methotrexate (MTX) was achieved through incubation of the nanoparticles (0.5 mg/mL) with a MTX solution for 12 h at room temperature under continuous agitation. Different drug loading concentrations were assessed (100, 250, and 500 μM). The non-encapsulated drug was removed through centrifugation (10,000 rpm, 20 min). The resulting MTX loaded nanoparticles (pellet) were redispersed in 1 mL of phosphate buffer solution (10 mM) at pH 7.4 for further analysis.

### 2.5. Preparation of Drug-Loaded Nanoparticle-Coated Contact Lenses

The loading of CLs either with MTX alone or with MTX-loaded CS/HA NPs, was performed by the method of immersion. All experiments were conducted in triplicate for each lens type.

For nanoparticle coating, CLs were immersed in a solution of CS/HA nanoparticles for 7 days and kept at 4 °C. On the 1st, 2nd, 3rd, and 7th days, 10 μL aliquots were collected, dried on a pre-weighted aluminum foil, and weighted to determine the adsorption efficiency of nanoparticles to CLs.

For the study of drug incorporation into CLs, a solution of MTX in ultrapure water was prepared, with the concentration of 500 μM of MTX. The CLs were soaked in the prepared solution for 2 days at a temperature of 4 °C.

For the assay of incorporation of MTX-loaded CS/HA nanoparticles into the CLs, CLs were immersed in a solution of drug-loaded CS/HA NPs with a concentration of 500 μM MTX for 2 days and kept at 4 °C.

The encapsulation efficiency (EE%) of methotrexate (MTX) within the NPs was assessed through fluorescence spectroscopy of the supernatant after centrifugation of the NPs (10,000 rpm, 20 min). Three independent measurements were performed for each system, and results are expressed as mean ± standard deviations (SD). The EE% and drug loading capacity (LC) were calculated using the following equations:

(1) $$EE\left(\%\right)=\frac{\left({drug}_{total}-{drug}_{non-encapsulated}\right)}{{drug}_{total}}\times 100$$

(2) $$LC\left(mg/mg\right)=\frac{\left({drug}_{total}(mg)-{drug}_{non-encapsulated}(mg)\right)}{\;nanoparticle\;content\;(mg)}\times 100$$

### 2.6. Drug Release Assays on CLs

Release assays were carried out following two adsorption conditions:

- CLs loaded with MTX-loaded nanoparticles
- CLs loaded with MTX alone.

For both conditions, the CLs were immersed in 1 mL of simulated tear environment (0.9% NaCl) and maintained at room temperature (25 ± 1 °C) under constant agitation of 380 rpm in closed dark glass vials covered with aluminum foil. The 0.9% NaCl solution was selected as a simplified and well-established in vitro release medium, commonly used in contact lens drug delivery assays, allowing reproducible sink conditions and direct comparison between formulations under controlled experimental variables.

I. Daily replacement contact lenses

In this type of lenses, release assays were carried out for 24 h from the moment in which they were placed in the solution (zero time). At pre-determined intervals (1, 2, 4, 6, 8, and 24 h), a 200 μL aliquot of the release medium was removed from the solution and analyzed by fluorescence spectroscopy. For every 200 μL removed, an equal volume (200 μL) of fresh solution was replaced in the release test, to keep sink conditions.

II. Monthly replacement contact lenses

In this type of lenses, the release tests were carried out over 31 days from the moment they were placed in the solution (0 days). After 1, 4, 5, 6, 7, 11, 12, 13, 19, 25, and 31 days, a 200 μL aliquot was removed from the solution, and the fluorescence spectrum was measured. For every 200 μL removed, an equal volume (200 μL) was replaced in the release test, to keep sink conditions.

Statistical analysis: SPSS software (Statistical Package for the Social Sciences, IBM SPSS Statistics, version 32.0.0, IBM Corp., Armonk, NY, USA) was employed for statistical analysis. The normality of the data distribution was tested using the Shapiro–Wilk test with Lilliefors correction. Normality of the variable distribution was inferred if the statistical significance value *p* > 0.05. Since the sample followed a normal data distribution, parametric tests were employed. The differences in means of quantitative variables for independent samples were compared using the Independent-Samples *t*-Test when the number of samples was two, and the One-Way ANOVA test when the number of samples was three or more. For multiple comparisons, the Bonferroni *post hoc* test was used when there was homogeneity of variances, and the Games-Howell *post hoc* test was used when there was no homogeneity of variances. Statistical significance was set at *p* < 0.05.

## 3. Results and Discussion

### 3.1. Chitosan/HA Nanoparticles Have Low Polydispersity and Long-Term Stability

**Figure 1:**
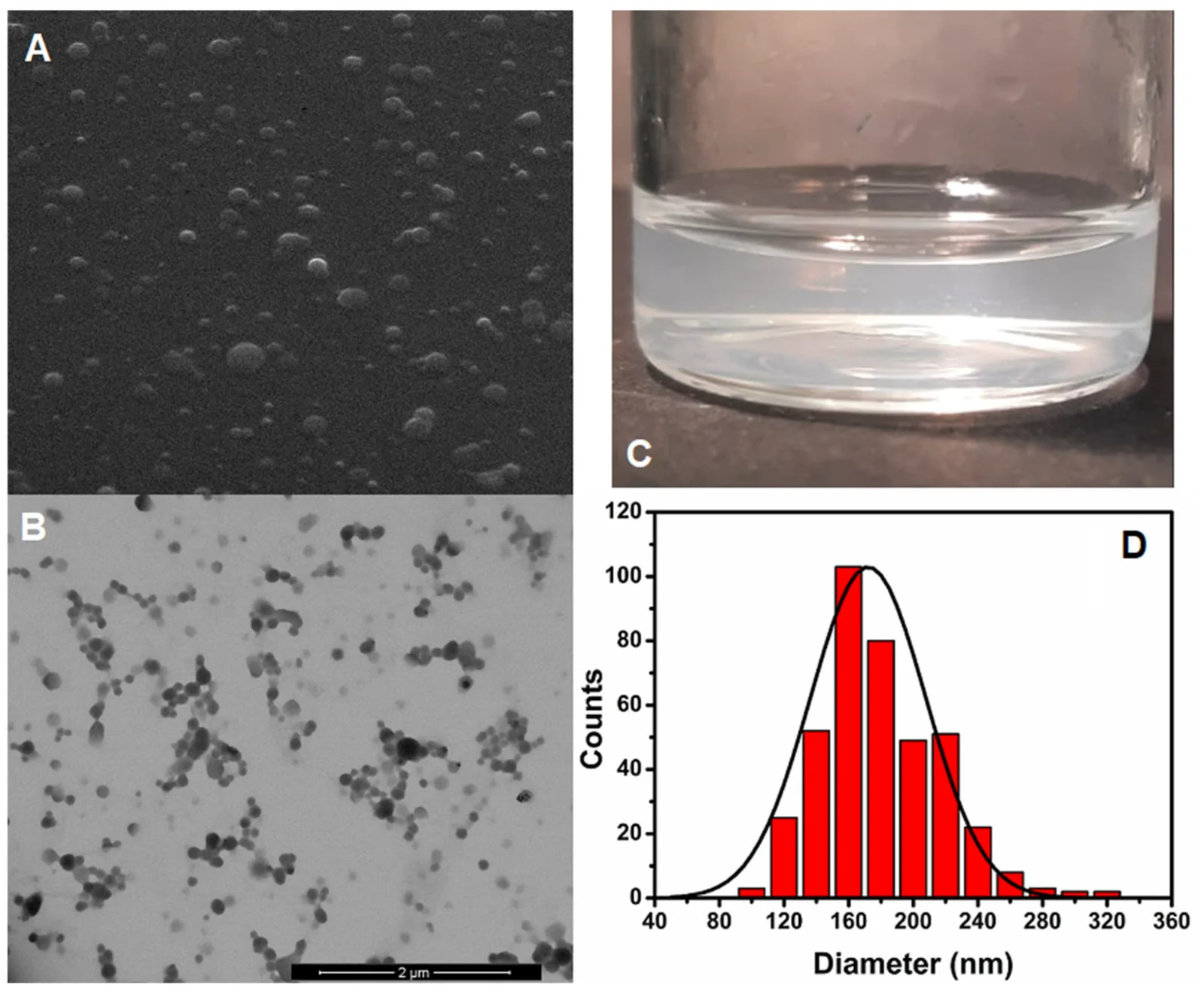
SEM (**A**) and STEM (**B**) images of the CS/HA nanoparticles, and (**D**) size distribution histogram of STEM image. (**C**) Aqueous dispersion of CS/HA nanoparticles at neutral pH.

Chitosan/Hyaluronic Acid (CS/HA) nanoparticles were prepared through ionotropic-gelation technique and coated with hyaluronic acid (HA) to obtain non-aggregated NPs with improved mucoadhesive properties (ensured by HA). After evaluating several CS/TPP different concentrations, the best formulation in terms of hydrodynamic diameter, low polydispersity (PDI) index and zeta potential was chosen (Formulation H, Table S1). This formulation presented a hydrodynamic diameter (270 ± 24 nm) suitable for biomedical applications, a very low polydispersity index (0.13 ± 0.04), and the most negative zeta potential (−12.8 ± 0.4 mV) among the studied formulations, indicating a lower tendency for aggregation. The size obtained here is somewhat smaller than that reported by Kalam (between 300 and 400 nm [19], with a lower PDI but also a less negative zeta potential. In agreement with other works [19,20], SEM and STEM images (Figure 1A,B) have revealed roughly spherical nanoparticles, with very few aggregated NPs. The NPs dispersion appears highly homogeneous (Figure 1C). The size histogram (Figure 1D) indicates smaller NPs (172 ± 36 nm), which was expected considering that these are dry particles, due to the vacuum conditions applied during the electron microscopy measurements.

**Figure 2:**
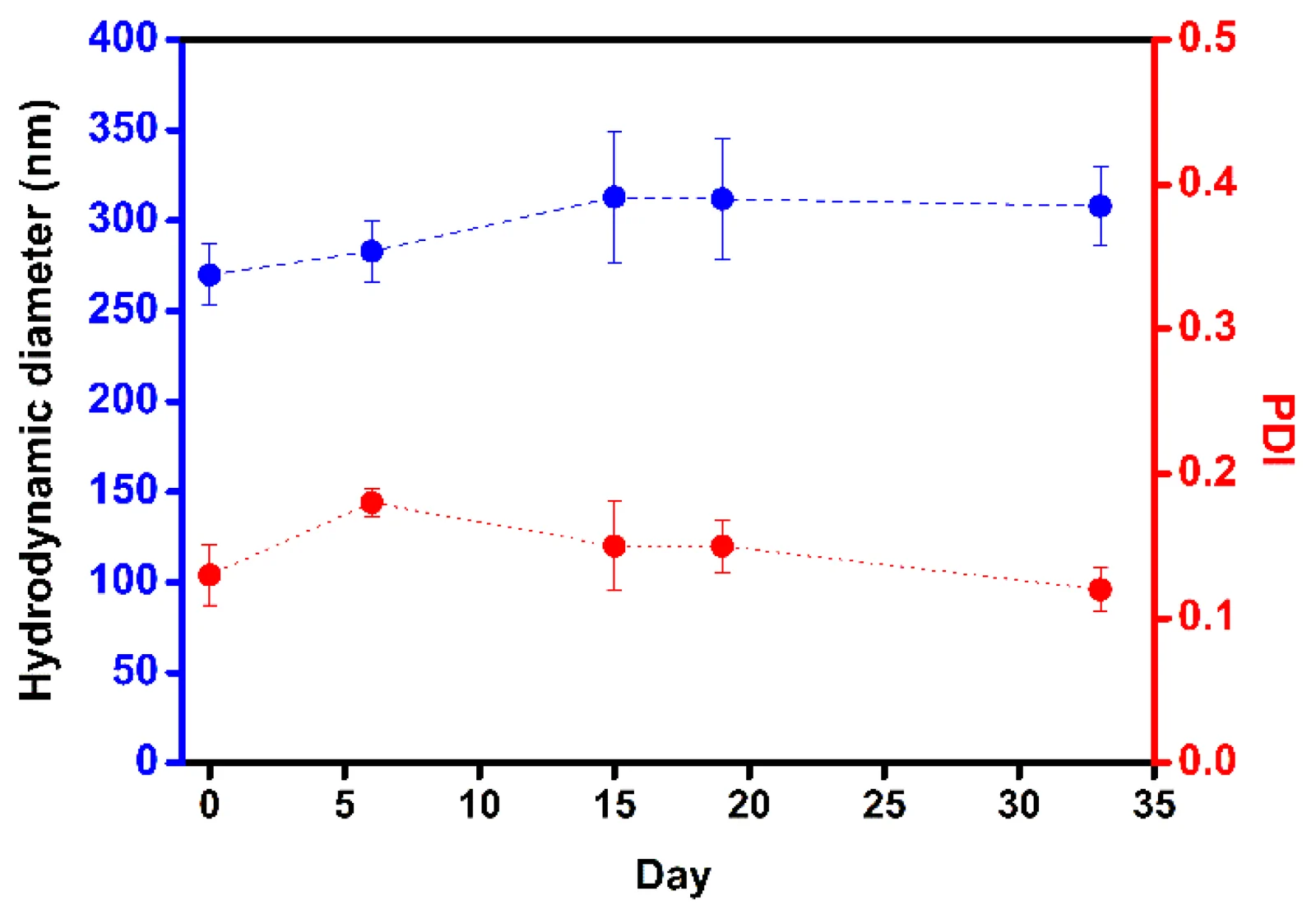
Variation in hydrodynamic diameter (blue) and polydispersity (PDI) value (red) for more than 30 days, for the chosen CS/HA NPs formulation at neutral pH, with storage at 4 °C.

Regarding stability of the chosen formulation, the variations in hydrodynamic diameter and PDI were followed for more than one month, during storage at 4 °C. The nanoparticles maintained their size, with only a very small increase, as well as the very low polydispersity values (Figure 2). This behaviour indicates a highly stable formulation, with suitable properties for application in monthly CLs. Notably, this storage stability at 4 °C is compatible with the conditions under which CLs are already commercially packaged and stored in sealed blister packs prior to use, suggesting that nanoparticle loading could potentially be integrated into this existing storage phase rather than requiring a separate industrial soaking step.

### 3.2. CS/HA Nanoparticles Are Suitable for MTX Encapsulation

Three different concentrations of MTX were tested for the loading of CS/HA NPs: 100, 250, and 500 μM, and the hydrodynamic diameter and PDI were determined (Table 2). There are no statistically significant differences (*p* > 0.05) in particle size parameter compared to the unloaded NPs, except between the 0 μM and 250 μM concentrations at pH 7.4.

Overall, the hydrodynamic diameter and PDI remained within a similar range for all tested concentrations, indicating that MTX incorporation did not significantly affect the nanoparticle size distribution. Also, zeta potential is roughly similar for all the MTX concentrations, with very small changes (from −11 mV at the lowest MTX concentration to ~−8 mV in the other cases), maintaining the negative surface charge, showing a negligible influence of MTX on the colloidal properties. This behaviour suggests that the CS/HA nanoparticle structure is able to accommodate MTX without inducing aggregation or major structural rearrangements, which is an important requirement for maintaining suitable physicochemical properties for ocular drug delivery.

**Figure 3:**
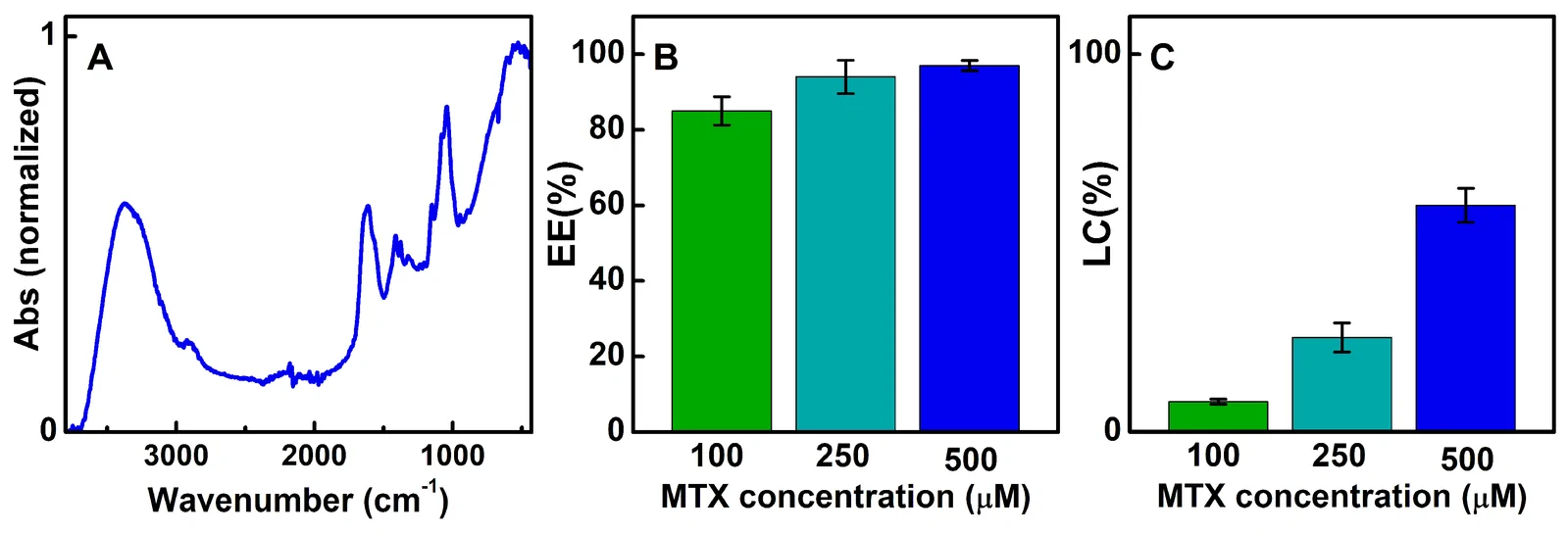
(**A**) FTIR spectrum of MTX-loaded CS/HA nanoparticles. (**B**) Encapsulation efficiency (EE%) and (**C**) loading capacity (LC%) of MTX in CS/HA nanoparticles. For EE% and LC%, three independent assays were performed. The error bars represent standard deviation (*n* = 3).

The FTIR spectrum of MTX-loaded CS/HA nanoparticles (Figure 3A) displays specific absorption bands corresponding to the functional groups of the materials involved. A wide band near 3400 cm^−1^ is linked to the contributions of O–H and N–H stretching vibrations. The presence of MTX within the nanoparticles is indicated by the presence of peaks near 1650 cm^−1^ and 1550 cm^−1^, corresponding to C=O stretching (amide I) and N–H bending (amide II), respectively. The C–O or P=O vibrations of the chitosan and TPP crosslinking, respectively, and the symmetric stretching of HA COO^−^ are linked to additional bands observed at 1400–1300 cm^−1^ and 1100 cm^−1^ [21,22,23,24]. These findings demonstrate that each component was successfully incorporated into the nanoparticles.

The encapsulation efficiency (EE%) of MTX in the NPs was analyzed using fluorescence spectroscopy (Figure 3B), through a previously determined calibration curve (fluorescence intensity vs. MTX concentration, see Supplementary Materials), for the three drug concentrations tested (100, 250 and 500 μM). The loading capacity (LC%) is displayed in Figure 3C. For all the MTX concentrations, the EE% exceeds 80%, the 500 μM concentration exhibiting the highest EE% value of (97 ± 1.3)%. This is also the concentration that provides the largest loading capacity (60 ± 4.5)%, while for the lower concentrations, LC% is (8 ± 0.7)% for 100 μM and (25 ± 3.9)% for 250 μM. Thus, the MTX concentration of 500 μM was chosen for the subsequent CLs assays.

The incorporation of CS/HA nanoparticles is expected to play an important role in modulating drug release behaviour. Encapsulation of MTX within the nanoparticles introduces an additional diffusion barrier, which may increase drug retention and slow its migration from the lens matrix into the surrounding medium. Chitosan-based systems are known to enhance mucoadhesion and promote sustained drug release due to electrostatic interactions and polymer swelling properties [24]. Moreover, conventional soaking methods used to load drugs into contact lenses often lead to rapid drug release and limited residence time on the ocular surface [3]. Thus, combining nanoparticle carriers with contact lens platforms represents a promising strategy to overcome these limitations and achieve a more sustained and controlled ocular drug delivery [3,24]. Importantly, this sustained interaction is not simply a polymer effect: it reflects the same mucoadhesive behaviour that hyaluronic acid and chitosan display in their native biological context, where HA mediates retention of the tear film over the corneal epithelium and chitosan-like polysaccharides interact with mucosal glycoproteins. The nanoparticle system developed here therefore functions as a biomimetic extension of this natural retention mechanism, applied to a synthetic drug carrier.

### 3.3. Drug-Laden and Nanoparticle-Laden CLs Are Advantageous as Delivery Systems

To test the efficiency of drug incorporation into CLs, two systems were compared: MTX loaded directly into CLs and MTX encapsulated in CS/HA NPs that were subsequently adsorbed into the CLs. The purpose of this comparison was to assess the potential advantages of the use of CS/HA NPs for the sustained release of MTX from CLs, considering that CLs have been widely reported as drug delivery systems [25,26,27,28].

**Figure 4:**
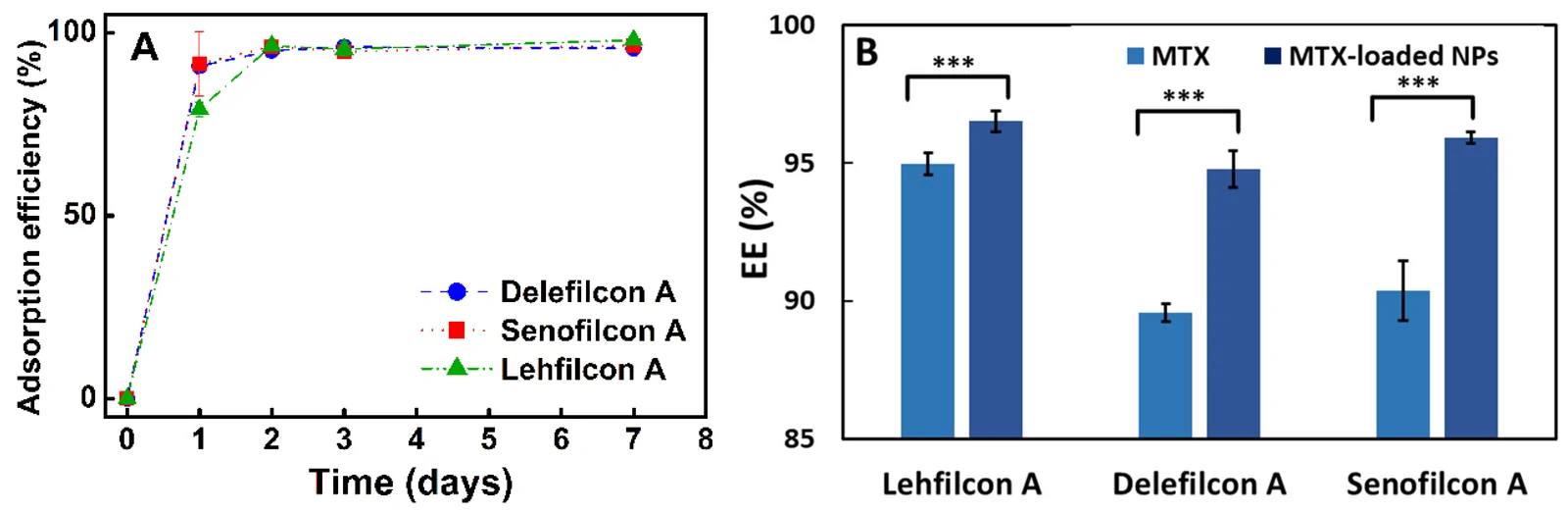
(**A**) Time dependence of nanoparticle adsorption efficiency of Senofilcon A, Delefilcon A and Lehfilcon A contact lenses. (**B**) Encapsulation efficiency of MTX in CLs. Error bars correspond to standard deviation. *** *p* ≤ 0.001. The error bars represent standard deviation (*n* = 3).

The immersion of CLs in drug-loaded nanoparticles’ solution was followed for seven days, reaching the saturation of nanoparticle adsorption after approximately two days. After that, the nanoparticles remained incorporated within the CLs. Despite the differences in water content and lens replacement modality (monthly or daily), Lehfilcon A (monthly disposable), Delefilcon A (daily disposable) and Senofilcon A (daily disposable), exhibited similar nanoparticle adsorption profiles (Figure 4A). This behaviour may be attributed to the high hydrophilicity and porosity of silicone hydrogel (Si-Hy) CLs [27,28] and emphasizes the high affinity between the CLs materials and the CS/HA NPs. This affinity is likely reinforced in the case of Delefilcon A, whose surface is based on a phosphorylcholine-containing material (MPC, Table 1) specifically designed to mimic the phospholipid bilayer of the cell membrane and the lubricating behaviour of the ocular glycocalyx [14]. This biomimetic surface chemistry, originally intended to improve lens wettability and comfort, may also favour the adsorption and retention of the CS/HA nanoparticles through similar hydration and interfacial mechanisms. The adsorption efficiency observed here is significantly higher than that previously reported for albumin/HA nanoparticles [10].

**Figure 5:**
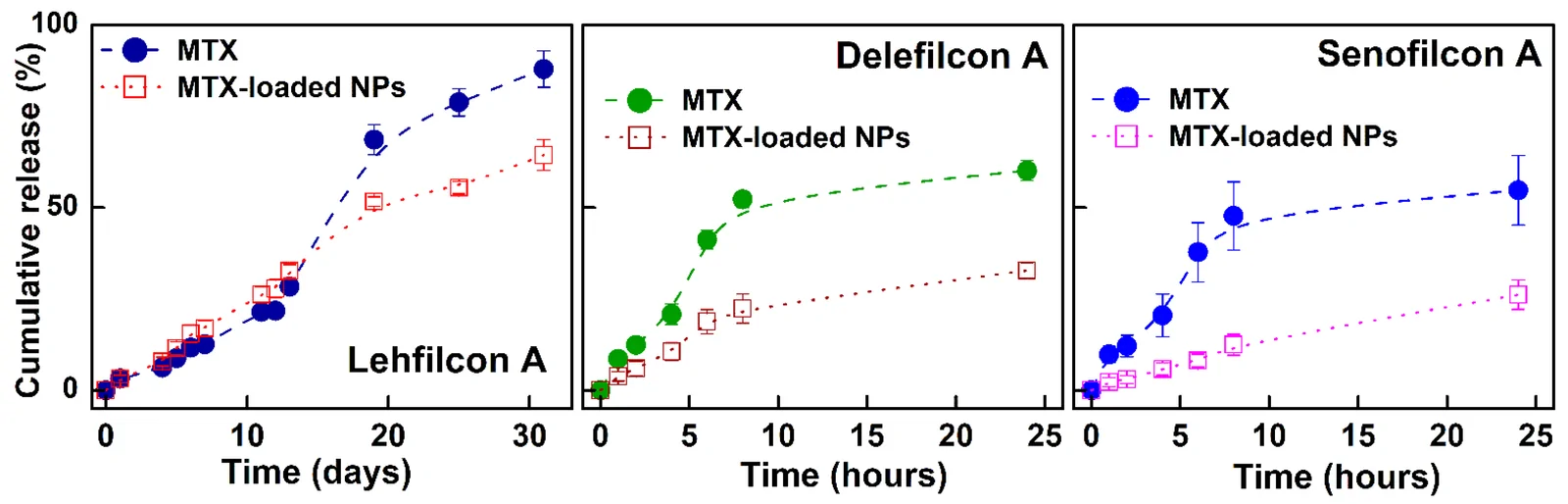
Release profiles of MTX from the different contact lenses, either from MTX-laden CLs or from CS/HA NPs loaded with MTX and incorporated into CLs in a simulated tear environment (0.9% NaCl). Error bars represent standard deviation from three independent assays (*n* = 3).

The CLs with incorporated MTX drug and those with MTX-loaded CS/HA nanoparticles were evaluated for drug release in a simulated tear environment (0.9% NaCl) to approach the ocular conditions [29]. The release profiles obtained for the three types of CLs are displayed in Figure 5, highlighting the more sustained release achieved with the NPs-loaded CLs compared to the drug-loaded CLs (without CS/HA nanoparticles). This behaviour can be attributed to the additional layer of resistance to drug diffusion promoted by the drug encapsulation within the nanoparticles [3], involving hydrogen bonding and electrostatic interactions between chitosan and methotrexate [30]. For instance, in the daily replacement lenses Senofilcon A and Delefilcon A, only 26% and 33% of the drug content was released after 24 h when delivered through nanoparticles, compared to 55% and 60%, respectively, when the drug was directly incorporated into the CLs.

The observed release profiles highlight the strong influence of CLs material properties on drug diffusion behaviour. Silicone hydrogel lenses differ in water content distribution, polymer architecture, and surface characteristics, which can significantly affect nanoparticle retention and drug transport pathways. In the present study, these material-dependent factors appear to influence the transition between burst release and sustained diffusion-controlled release mechanisms. The more hydrophobic surface of Senofilcon A (contact angle 50°) is consistent with its slower release compared with the more hydrophilic Delefilcon A (contact angle 34°, 100% surface water content), suggesting that surface hydrophilicity facilitates faster initial drug diffusion in daily lenses. Lehfilcon A, however, combines the highest core water content (55%) with the most hydrophilic surface (contact angle 25°) among the three materials, yet shows the slowest overall release. This apparent contrast suggests that, for this material, release is governed less by water content and more by the polymer network architecture (Tris/HEMA/NVP chemistry) and crosslinking density typical of monthly replacement lenses, in line with the swelling/relaxation-controlled mechanism identified by the Korsmeyer–Peppas model (*n* ≥ 0.85).

The initial rapid release observed in daily replacement lenses is consistent with drug diffusion primarily occurring from superficial regions of the lens matrix, where weakly associated drug or nanoparticles can be more readily released into the surrounding medium. Similar burst release behaviour has been previously reported in drug-loaded hydrogel lenses and is commonly attributed to limited drug-polymer interactions during passive loading approaches [3,31].

Additional insight on drug release mechanisms can be obtained by fitting the profiles to suitable mathematical models. In this work, different models were used to analyze the data, namely Korsmeyer–Peppas, [32] Gompertz [33] and Weibull models [34] (details of the models in Supporting Information). In the case of the Korsmeyer-Peppas model, the one that better fits the experimental results, parameter *n* is associated with the mechanism of drug diffusion (Table S2 in Supplementary Materials). In general, the profiles could be explained by an anomalous transport, considering the values of the exponent *n* between 0.43 and 0.85 (case II transport). However, for the monthly Lehfilcon A CLs, *n* ≥ 0.85, indicating that the release is mainly driven by swelling or relaxation of network chains. While the Gompertz model does not fit the experimental results well, the Weibull model allows us to predict a complex release mechanism for the monthly CLs and the daily Delefilcon A (*b* > 1), with the exception of Senofilcon A incorporating drug-loaded nanoparticles, where a combined mechanism (Fickian diffusion and case II transport) is predicted by this model (0.75 < *b* < 1).

Overall, the three CL materials are suitable for methotrexate release, while the incorporation of MTX-loaded CS/HA nanoparticles allows a more controlled and sustained drug release profile.

In particular, the monthly replacement lens (Lehfilcon A) demonstrated a markedly slower and more sustained release profile, suggesting enhanced nanoparticle retention within the lens structure. This behaviour may be associated with differences in polymer composition and internal morphology, which can increase diffusion path length and reduce drug mobility. The reduced initial drug loss (<5%) further supports the hypothesis of stronger interactions between the nanoparticle system and the lens matrix.

Extended release profiles are particularly desirable for chronic ocular therapies, as sustained drug availability may reduce dosing frequency while maintaining therapeutic levels at the ocular surface. Previous studies have shown that the increasing of drug-material interactions within silicone hydrogel lenses represents a key strategy for achieving prolonged delivery kinetics [4]. In particular, topical MTX has demonstrated therapeutic potential for inflammatory dry eye disease when administered as eye drops at 1 mg/mL four times daily over a follow-up period of six to eight weeks [12]. Although the present study was not designed to establish dose equivalence with topical eye drop regimens, the sustained release obtained for the CS/HA nanoparticle-laden CLs formulations supports their potential as platforms for prolonged ocular drug delivery. Based on the MTX concentration used during nanoparticle loading, together with the high adsorption efficiency to the lenses, the theoretical MTX content per lens can be estimated to ~0.2 mg. In this way, given the low bioavailability of topical eye drops (<5%) [1,2], the prolonged release of MTX on the ocular surface for extended periods via chitosan/hyaluronic acid nanoparticle-laden CLs may represent an advantageous delivery strategy.

### 3.4. Drug-Laden and Nanoparticle-Laden CLs Maintain the Optical and Structural Properties of Neat CLs

To assess the influence of the drug and the drug-loaded nanoparticles in the CL properties, the refractive index, UV/Visible transmittance and FTIR spectra were measured and compared to those of the neat CLs.

**Figure 6:**
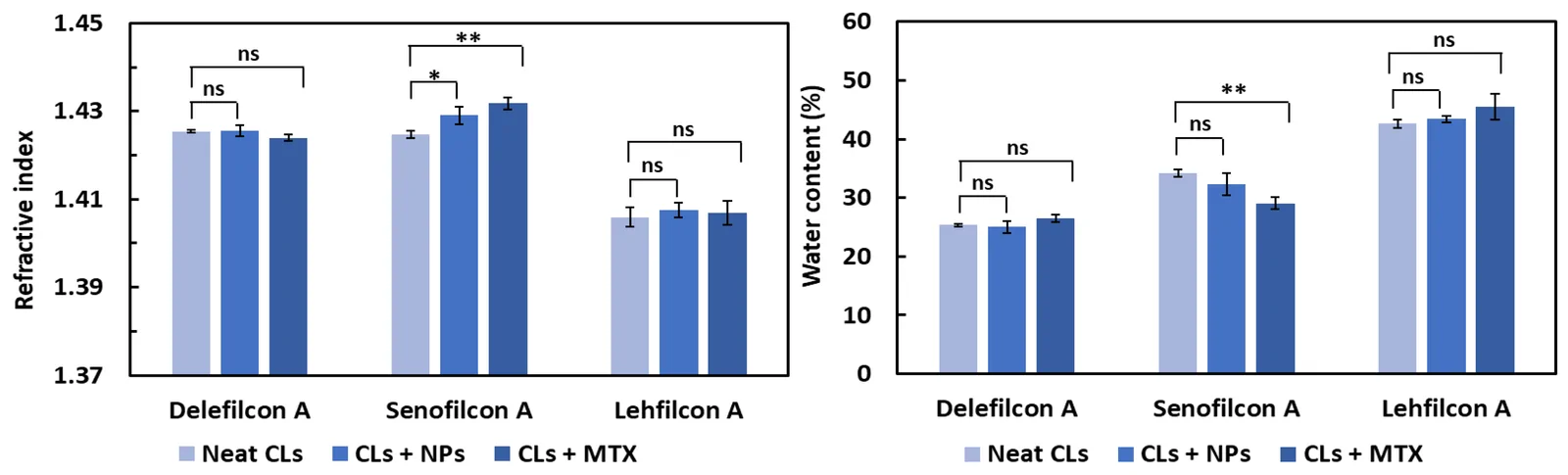
Refractive index (**left**) and water content (**right**) of neat CLs, CLs with adsorbed NPs and CLs with incorporated MTX. Error bars correspond to standard deviation; ns: non-significant (*p* > 0.05), (*) *p* ≤ 0.05, and (**) *p* ≤ 0.01.

Figure 6 shows the refractive index (left) and associated water content (right) for the three lenses’ materials. Only in the case of Senofilcon A, statistically significant differences were observed in refractive index and water content, with the latter decreasing slightly for the drug-laden CLs. The slight decrease in water content and corresponding increase in refractive index observed in Senofilcon A lenses loaded with MTX suggests minimal interaction of the additives with the hydrogel matrix, without compromising oxygen permeability or lens hydration.

**Figure 7:**
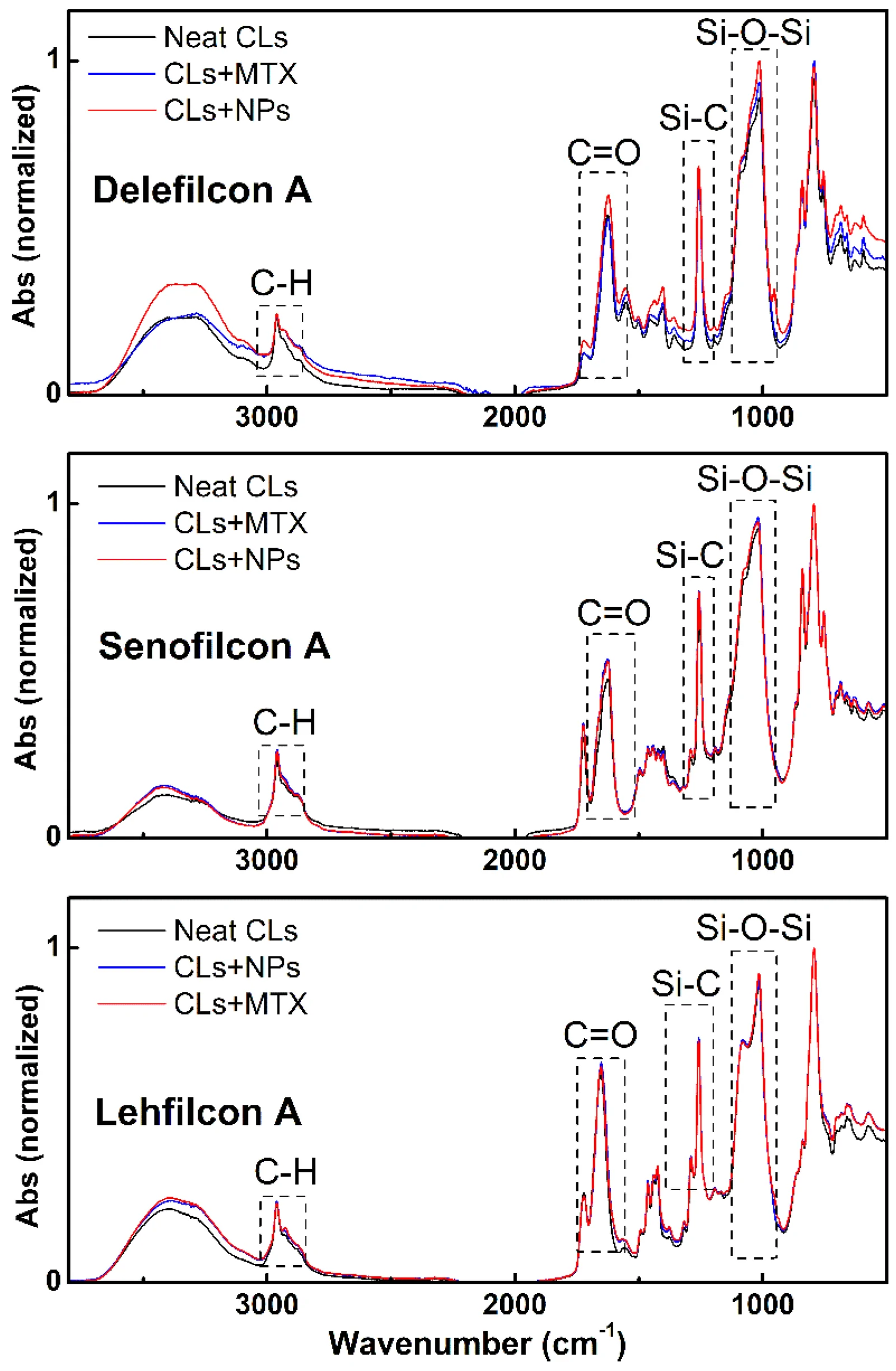
Fourier transform infrared (FTIR) spectra of the three types of CLs, Delefilcon A, Senofilcon A, Lehfilcon A. For each material, neat CLs, CLs with adsorbed NPs and CLs with incorporated MTX were analyzed.

FTIR spectra (Figure 7) were highly similar across all samples, particularly in the main characteristic peaks of the CL material. The absorption bands, including those around 3300 cm^−1^ (O–H/N–H stretching), 2900 cm^−1^ (C–H stretching), 1650 cm^−1^ (C=O stretching), and 1000–1100 cm^−1^ (Si–O–Si or C–O stretching), are evident in the three samples. No significant changes in the major peak wavelengths are observed, indicating that the polymeric structure of the CLs remains chemically stable upon the incorporation of nanoparticles and methotrexate.

In this sense, FTIR analysis confirmed that the polymeric backbone of all three CL materials remained intact, supporting the chemical stability of the lenses upon drug or nanoparticle loading.

**Figure 8:**
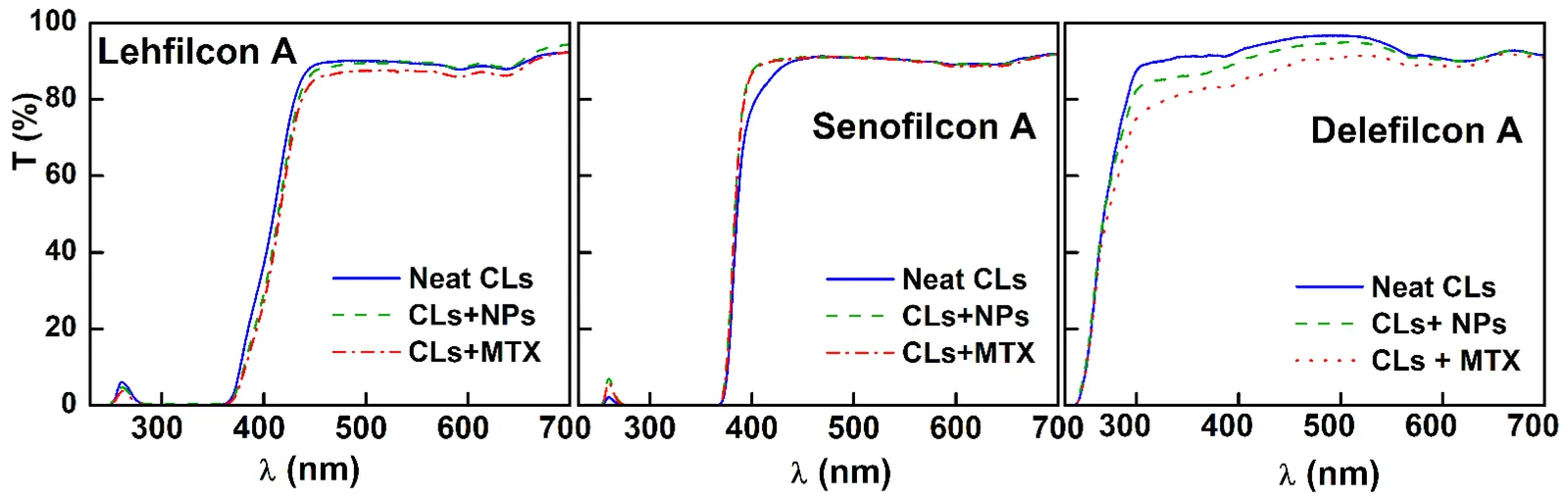
UV/Visible transmittance spectrum of neat CLs, CLs with adsorbed NPs and CLs with incorporated MTX.

Optical transmittance is also an essential property when considering CL wear. Figure 8 displays transmittance spectra in the UV/Visible region for the three CL materials. Senofilcon A and Lehfilcon A display typical spectra for CLs with UV-blocking filter. In the visible region, all the CLs display transmittance percentage (%T) values around or above 90%, with a small decrease (around 5%) for MTX-laden Delefilcon A CLs. These results show that loaded CLs, either with only MTX or with drug-loaded CS/HA nanoparticles, maintain a high and mostly unaffected optical transmittance in the visible region of the spectrum. Moreover, the UV-blocking filters also maintain their efficiency in drug-laden and nanoparticle-laden CLs.

The preserved optical transmittance in the visible region (>90%) for all loaded lenses indicates that neither MTX nor CS/HA nanoparticles compromise lens transparency, while UV-blocking properties remain effective.

Differences in water content, polymer composition, and silicone/hydrogel ratio among the three lens materials appear to influence both nanoparticle retention and drug diffusion, highlighting the role of lens material as an active parameter in designing sustained ocular delivery systems.

From a clinical perspective, the distinct release behaviours observed between daily and monthly replacement lenses may enable therapy personalization according to treatment duration requirements. Rapid drug release from daily lenses may be advantageous for short-term anti-inflammatory treatments, whereas sustained delivery from monthly lenses could support long-term management of chronic ocular diseases. In this sense, these results support the potential of nanoparticle-CL systems as adaptable platforms for ocular drug delivery, enabling treatment strategies tailored to short- or long-term therapeutic needs. In addition, the results reinforce the importance of considering lens material selection as an active design parameter in the development of therapeutic CLs, rather than treating lenses as passive drug carriers.

Despite promising results, several limitations should be considered. Release experiments were conducted under in vitro conditions, which do not fully reproduce the dynamic ocular environment, including tear turnover, blinking, protein deposition, and mechanical stresses associated with lens wear. These factors may influence nanoparticle retention and drug diffusion behaviour in vivo. Constant immersion may overestimate drug availability compared with physiological tear volumes. In addition, the mechanical properties of the lenses (e.g., tensile modulus) after nanoparticle soaking were not evaluated in this study, and changes in these properties could affect wearer comfort and corneal integrity. Biological response of ocular tissues and potential long-term biocompatibility effects associated with repeated exposure to nanoparticle-loaded lenses were not assessed and require further investigation. A limitation of this study is the lack of in vitro cytotoxicity assays with corneal epithelial cells; although the MTX concentrations released are expected to be well below those linked to corneal toxicity in intravitreal use, and topical MTX in the low mg/mL range has shown good short-term corneal epithelial cell viability and clinical tolerability in ocular surface disease [12], such safety assessments remain essential and will be pursued in subsequent studies. Future studies involving ex vivo or in vivo models, together with mechanical and cytotoxicity characterization, as well as long-term chemical stability and biological activity, will be necessary to confirm therapeutic efficacy, safety, and clinical performance under real wearing conditions. Overall, the present approach illustrates how therapeutic CLs can move beyond passive drug reservoirs by drawing on biomimetic design principles. By combining nanocarriers composed of chitosan and hyaluronic acid, materials that echo the natural lubrication and mucoadhesive function of the tear film, with silicone hydrogel matrices engineered to replicate the hydration behaviour of the ocular surface, this platform brings the synthetic device closer to the biological environment it is meant to serve, an aspect that may prove relevant for future generations of ocular drug delivery systems.

## 4. Conclusions

This study demonstrates that lens material and replacement modality strongly influence methotrexate release kinetics from nanoparticle-laden silicone hydrogel lenses. Daily replacement lenses released 26 ± 4% (Senofilcon A) and 33 ± 5% (Delefilcon A) of MTX after 24 h, respectively. The monthly lens (Lehfilcon A) showed slower, diffusion-controlled release, with 15 ± 2% released at 24 h and 64 ± 3% cumulative release after 31 days (*p* < 0.01). Incorporation of CS/HA nanoparticles improved MTX retention and allowed modulation of the release behaviour compared with lenses loaded with the MTX alone. These findings support CS/HA nanoparticle-laden silicone hydrogel contact lenses as tunable platforms for ocular drug delivery, with daily lenses suited to short-term treatments and monthly lenses to sustained, long-term therapy.

## Figures and Tables

**Table 1:** Characteristics of the contact lenses as reported by the manufacturer.

USAN	Lehfilcon A	Delefilcon A	Senofilcon A
Manufacturer	Alcon	Alcon	Johnson & Johnson
Commercial name	Total 30^TM^	DAILIES TOTAL1™	Acuvue^®^ Oasys 1-Day with HydraLuxe^TM^
Water content (%)	55 (core); 100 (surface)	33 (core); 100 (surface)	38
Dk/t (units) (−3.00 D)	154	156	121
Central thickness (mm) (−3.00 D)	0.080	0.090	0.085
Base curve (mm)	8.40	8.50	8.50
Diameter (mm)	14.20	14.10	14.30
US-FDA group	V	V	I
Filter	UV—class I, HEVL	No	UV—class I
Replacement	Monthly	Daily	Daily
Main monomers	Tris/HEMA/NVP	MPC [16]	mPDMS/DMA/HEMA/siloxane macromer/PVP/TEGDMA [17]
Contact angle	25°	34° [16]	50°
Surface treatment	No	No	No

USAN: United States Adopted Name. DK/t: oxygen transmissibility (units: ×$10^{-9}$ (cm/s) (mL O_2_/mL·mm Hg)). HEVL: high-energy visible light; mPDMS: polydimethylsiloxane monofunctional; DMA: *N*,*N*-dimethyl-acetamide; HEMA: hydroxyethyl methacrylate; PVP: polyvinyl pyrrolidone; TEGDMA: tetraethyleneglycol dimethacrylate; MPC: 2-methacryloyloxyethyl phosphorylcholine; Tris: 3-[Tris(trimethylsiloxy)silyl]propyl methacrylate; NVP: *N*-Vinylpyrrolidone.

**Table 2:** Results of the DLS analysis of CS/HA nanoparticles loaded with three concentrations of MTX (100, 250, and 500 μM) at pH 7.4. All comparisons are relative to the non-loaded nanoparticles (0 μM). ns: non-significant.

MTX Conc. (μM)	Hydrodynamic Diameter ± SD (nm)	Statistical Significance	PDI	Statistical Significance	Zeta Potential (mV)	Statistical Significance
100	289 ± 20	ns	0.14	ns	−11.08	ns
250	218 ± 24	*p* ≤ 0.05	0.21	ns	−7.97	ns
500	284 ± 16	ns	0.13	ns	−8.53	ns

## Data Availability

The original contributions presented in this study are included in the article and its Supplementary Materials. Further inquiries can be directed to the corresponding authors.

## Supplementary Materials

The following supporting information can be downloaded at: https://www.mdpi.com/article/10.3390/biomimetics11080532/s1, Table S1: Hydrodynamic diameter, PDI index and zeta potential values of formulations with varying Chitosan and TPP concentrations, in 10 mM phosphate buffer (pH = 7.4), keeping constant the hyaluronic acid concentration. The chosen formulation is highlighted.; Figure S1: (A) Normalized fluorescence spectra of MTX in aqueous medium immediately after solution preparation (blue curve) and released from monthly CLs after 31 days (red curve), showing no MTX degradation. (B) Calibration curve of MTX fluorescence intensity in function of concentration; Drug release assays; Table S2: Release coefficients of the Korsmeyer–Peppas, Gompertz and Weibull models obtained from the fitting of the release profiles of MTX.
