# Supplementary material for: Contact Lenses Incorporating Methotrexate-Loaded Chitosan/Hyaluronic Acid Nanoparticles
Source: Biomimetics (Basel). 2026 Aug 1;11(8):532. doi: 10.3390/biomimetics11080532 (PMC13509337; doi:10.3390/biomimetics11080532)
Supplement: Supplementary file 1 [file biomimetics-11-00532-s001.zip › biomimetics-4424252-supplementary.pdf]

Contact Lenses Incorporating Methotrexate-Loaded Chitosan/Hyaluronic Acid Nanoparticles

Sofia Vale [1,2], Sara F. M. Senra [1,2], Sérgio R. S. Veloso [3], Elisabete M. S. Castanheira [1,2,*] and Madalena Lira [1,2,*]

[1] Physics Centre of Minho and Porto Universities (CF-UM-UP), University of Minho, Campus de Gualtar, 4710-057 Braga, Portugal; pg50962@alunos.uminho.pt (S.V.)
[2] LaPMET Associate Laboratory, University of Minho, Campus de Gualtar, 4710-057 Braga, Portugal
[3] CINBIO, University of Vigo, 36310 Vigo, Spain; sergiorafael.dasilva@uvigo.gal
* Correspondence: ecoutinho@fisica.uminho.pt (E.M.S.C.); mlira@fisica.uminho.pt (M.L.)

Supplementary Materials

Table S1. Hydrodynamic diameter, PDI index and zeta potential values of formulations with varying Chitosan and TPP concentrations, in 10 mM phosphate buffer (pH=7.4), keeping constant the hyaluronic acid concentration. The chosen formulation is highlighted.

Formulation	TPP conc. (wt%)	CS conc. (w%)	HA conc. (wt%)	Hydrodynamic diameter (nm) ± SD	PDI ± SD	Zeta potential (mV) ± SD
**A**	0.06	0.025	0.05	373 ± 31	0.12 ± 0.08	-10.32 ± 0.11
**B**	0.06	0.050	0.05	230 ± 34	0.19 ± 0.02	-7.53 ± 0.5
**C**	0.06	0.075	0.05	274 ± 23	0.17 ± 0.06	-6.38 ± 0.2
**D**	0.12	0.025	0.05	358 ± 22	0.19 ± 0.03	-9.72 ± 0.12
**E**	0.12	0.050	0.05	329 ± 7	0.12 ± 0.05	-7.18 ± 0.09
**F**	0.12	0.075	0.05	293 ± 4	0.20 ± 0.04	-7.35 ± 0.2
**G**	0.25	0.025	0.05	283 ± 35	0.13 ± 0.09	-9.71 ± 0.20
**H**	0.25	0.050	0.05	270 ± 24	0.13 ± 0.04	-12.8 ± 0.4
I	0.25	0.075	0.05	232 ± 3	0.12 ± 0.02	-9.34 ± 0.20

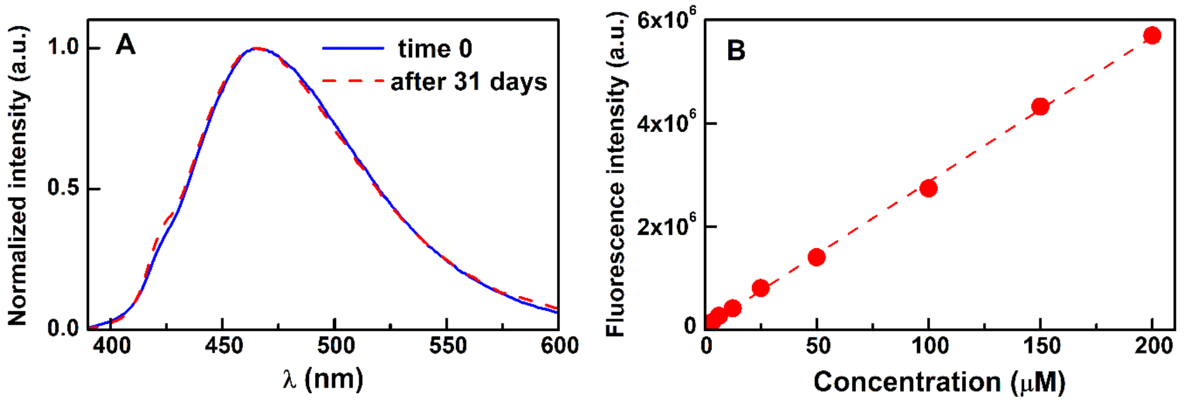

Figure S1. (**A**) Normalized fluorescence spectra of MTX in aqueous medium immediately after solution preparation (blue curve) and released from monthly CL after 31 days (red curve), showing no MTX degradation. (**B**) Calibration curve of MTX fluorescence intensity in function of concentration.

Drug release assays

The Gompertz and Korsmeyer-Peppas models are, respectively, described according to the equations:

$$X_t = X_{max} e^{-ae^{b \log_{10} t}} \quad \text{(S1)}$$

$$\frac{M_t}{M_\infty} = K_s t^n \quad \text{(S2)}$$

in which $\frac{M_t}{M_\infty}$ is the fraction of drug released at time *t*, and K_s is the rate constant. For a spherical geometry, when $n < 0.43$, the release mechanism is diffusion-controlled (Fickian diffusion), $0.43 < n < 0.85$ is an anomalous transport, and $n \geq 0.85$ indicates that the release is mainly driven by swelling or relaxation of network chains (case-II transport). The X_t and X_{max} are the dissolved drug fractions at time t and its maximum, a is a shape parameter and b is the dissolution rate per unit of time.

The Weibull model is a distribution function, which expresses the compound fraction accumulated (m) in solution at time t, according to Equation (S3),

$$m = 1 - \exp\left[\frac{-(\mathrm{t} - \mathrm{T}_i)^b}{a}\right] \quad \text{(S1)}$$

where *a* is a parameter defining the timescale of the process, T_i is a location parameter representing the latency time of the release mechanism (considered zero many times), and *b* denotes the curve type shape parameter. For $b > 1$, the transport follows a complex release mechanism; $b \leq 0.75$

indicates Fickian diffusion (in either fractal or Euclidian spaces) and 0.75 < *b* < 1 indicates a combined mechanism (Fickian diffusion and Case II transport).

Table S2. Release coefficients of the Korsmeyer-Peppas, Gompertz and Weibull models obtained from the fitting of the release profiles of MTX.

CL	System	Korsmeyer-Peppas			Gompertz				Weibull		
		K_S (h^{-1})	n	R^2	X_{max}	a	b	R^2	a	b	R^2
**Delefilcon A**	MTX@CLs	0.082	0.662	0.999	1	2.66	1.18	0.922	0.048	1.30	0.989
	MTX-NP@CLs	0.039	0.677	0.998	1	3.37	0.83	0.980	0.031	1.03	0.992
**Senofilcon A**	MTX@CLs	0.089	0.588	0.996	1	2.62	1.17	0.941	0.057	1.16	0.988
	MTX-NP@CLs	0.035	0.783	0.998	1	4.95	0.93	0.981	0.019	0.86	0.994
**Lehfilcon A**	MTX@CLs	1.3×10^{-3}	1.24	0.983	1	3×10^{5}	5.09	0.932	3×10^{-4}	1.02	0.984
	MTX@NP@CLs	8.4×10^{-4}	1.03	0.995	1	161	2.02	0.970	1.6×10^{-4}	1.17	0.990
